# Signatures of Selection on Mitonuclear Integrated Genes Uncover Hidden Mitogenomic Variation in Fur Seals

**DOI:** 10.1093/gbe/evac104

**Published:** 2022-07-09

**Authors:** David L J Vendrami, Toni I Gossmann, Nayden Chakarov, Anneke J Paijmans, Vivienne Litzke, Adam Eyre-Walker, Jaume Forcada, Joseph I Hoffman

**Affiliations:** Department of Animal Behaviour, Bielefeld University, 33501 Bielefeld, Germany; Department of Animal Behaviour, Bielefeld University, 33501 Bielefeld, Germany; Department of Animal Behaviour, Bielefeld University, 33501 Bielefeld, Germany; Department of Animal Behaviour, Bielefeld University, 33501 Bielefeld, Germany; Department of Animal Behaviour, Bielefeld University, 33501 Bielefeld, Germany; School of Life Science, University of Sussex, Brighton BN1 9QG, UK; British Antarctic Survey, High Cross, Madingley Road, Cambridge CB3 OET, UK; Department of Animal Behaviour, Bielefeld University, 33501 Bielefeld, Germany; British Antarctic Survey, High Cross, Madingley Road, Cambridge CB3 OET, UK

**Keywords:** nuclear DNA sequences of mitochondrial origin (*numts*), genome evolution, mitochondrial DNA, pinniped, Antarctic fur seal, *Arctocephalus gazella*

## Abstract

Nuclear copies of mitochondrial genes (*numts*) are commonplace in vertebrate genomes and have been characterized in many species. However, relatively little attention has been paid to understanding their evolutionary origins and to disentangling alternative sources of insertions. *Numts* containing genes with intact mitochondrial reading frames represent good candidates for this purpose. The sequences of the genes they contain can be compared with their mitochondrial homologs to characterize synonymous to nonsynonymous substitution rates, which can shed light on the selection pressures these genes have been subjected to. Here, we characterize 25 *numts* in the Antarctic fur seal (*Arctocephalus gazella*) genome. Among those containing genes with intact mitochondrial reading frames, three carry multiple substitutions in comparison to their mitochondrial homologs. Our analyses reveal that one represents a historic insertion subjected to strong purifying selection since it colonized the Otarioidea in a genomic region enriched in retrotransposons. By contrast, the other two *numts* appear to be more recent and their large number of substitutions can be attributed to noncanonical insertions, either the integration of heteroplasmic mtDNA or hybridization. Our study sheds new light on the evolutionary history of pinniped *numts* and uncovers the presence of hidden sources of mitonuclear variation.

**Significance**

Eukaryotic organelles, such as mitochondria or plastids, possess their own genomes. Intriguingly, fragments of organelle DNA can be found in the nuclear genomes of many species. These are known as *numts* and represent mitochondrial DNA that has been copied into a nuclear genome. These insertions are assumed to be nonfunctional and little is known about their evolutionary fates. We conducted a genome-wide scan of *numts* in the Antarctic fur seal genome and found one *numt* that has been subjected to strong purifying selection. We further identified two *numts* that appear to have originated from noncanonical sources. Our study shows that *numts* can have complex evolutionary histories and that they need not necessarily be nonfunctional.

## Introduction

Mitochondria are organelles that are believed to share a common ancestor with α-protobacteria (Sagan 1967). During their evolution as endosymbionts of eukaryotic cells, their genomes dramatically reduced in size as the vast majority of their functional genes transferred into the nucleus over the last 1.5 billion years (Bock and Timmis 2008). Mitochondrial colonization of the nuclear genome is a continuous process that is still ongoing (Ricchetti et al. 2004). This is reflected by the presence of numerous nuclear sequences revealing homology to contemporary mitochondrial DNA (mtDNA), usually referred to as “nuclear mtDNA sequences” or *numts*, across a wide range of eukaryotic species (Hazkani-Covo et al. 2010; Calabrese et al. 2017).

With the increasing number of available reference genomes, considerable effort has been invested over the past decade into characterizing *numts*, especially in mammalian species (Calabrese et al. 2017; Schiavo et al. 2017; Grau et al. 2020). These studies have shown that the number of *numts* present in a nuclear genome can vary substantially across species, from as few as 13 *numts* in the polar bear (*Ursus maritius*) (Lammers et al. 2017) to over 1,000 in the opossum (*Monodelphis domestica*) (Hazkani-Covo et al. 2010) and platypus (*Ornithorhynchus anatinus*) (Calabrese et al. 2017). However, limited attention has so far been paid to understanding whether *numts* could be functional or to distinguishing among possible alternative sources of mitonuclear integrations.

It has been shown that certain *numts*, depending on their insertion position, can have deleterious effects and be associated with diseases (Borensztajn et al. 2002; Turner et al. 2003). Yet, *numts* do not appear to be functional *per se*, as most of them become pseudogenes upon their integration into the nuclear genome due to the presence of premature stop codons resulting from the difference between the nuclear and mitochondrial genetic codes (Bensasson et al. 2001). After their insertion into the nuclear genome, *numts* are then expected to accumulate mutations over time, causing their mitochondrial reading frame to quickly degrade. *Numts* deviating from this assumption, namely ancient mitonuclear insertions (i.e. insertions predating the split between major taxonomic groups such as orders) presenting intact mitochondrial reading frames despite the presence of numerous synonymous mutations, could then be seen as potential evidence for a *numt* sequence being under functional constraint. Although it is unlikely that most *numts* are expressed (Bensasson et al. 2001) or play a role in RNA–RNA interactions (Cloonan 2015), they could nevertheless exercise a functional role as regulatory elements or through the creation of novel exons (Noutsos et al. 2007).

As described above, *numts* that carry numerous mutations in comparison to their mitochondrial homologs may intuitively be regarded as ancient insertions, because they will have had more time to diverge. However, appreciable numbers of substitutions might also be observed for more recent insertions (i.e. insertions that occurred after the split between major taxonomic groups and that are observed only in a single species or genus) when they derive from noncanonical sources, such as heteroplasmic mitochondrial variants (Wei et al. 2020; Formenti et al. 2021; Parakatselaki and Ladoukakis 2021) or mtDNA that has introgressed via hybridization (Baldo et al. 2011). Genes residing in these recent insertions might be expected to contain intact mitochondrial reading frames because they were functional in the mitochondrion before their recent insertion into the nuclear genome. By contrast, assuming that *numts* are selectively neutral, ancient insertions should have a greater likelihood of containing genes with premature stop codons and/or out of frame mutations, as defined by the mitochondrial genetic code.

To identify *numts* that are possibly functional and/or which may have originated from alternative sources, one could search for genes residing in mitonuclear insertions that show intact mitochondrial reading frames and compare them to their mitochondrial homologs. Intact mitochondrial reading frames in *numts* containing mutations would then be indicative of either ancient insertions that violate the assumption of *numts* being nonfunctional or recent insertions from alternative sources. The amounts of synonymous substitutions (dS), together with phylogenetic analysis, could then be used to distinguish recent mitonuclear insertions from ancient ones. Finally, the ratio of synonymous and nonsynonymous substitutions (dN/dS) could be analyzed to reveal the evolutionary pressures that these potentially coding sequences have been subjected to Kryazhimskiy and Plotkin (2008) and to formally test for the presence of nonneutral *numts*.

The Antarctic fur seal (*Arctocephalus gazella*) represents an ideal candidate to search for *numts* that might contain functional genes and/or genes that have originated from noncanonical sources. First, *A. gazella* is known to have recently hybridized with other two *Arctocephalus* species (Lancaster et al. 2006). This may have introduced novel mitochondrial variation that, if integrated into the nuclear genome, would appear divergent from homologous *A. gazella* mitochondrial sequences. Second, both a high quality, chromosome-level, nuclear reference genome (Humble et al. 2018, 2016; Peart et al. 2021) and a mitochondrial (Nagel et al. 2019) genome are available for this species.

Here, we performed a scan of the nuclear reference genome of the Antarctic fur seal for mitonuclear insertions, identifying a total of 25 *numts*. Four of these contain genes with intact mitochondrial reading frames. Among those containing mutations, one appears to be an ancient insertion exhibiting strong signatures of purifying selection, whereas two *numts* appear to represent recent integrations of highly divergent mtDNA sequences. Taken together, our results provide new insights into the evolutionary forces shaping *numts* in pinnipeds and show how the study of mitonuclear genes with intact mitochondrial reading frames can uncover hidden sources of mitonuclear variation.

## Results

### Discovery and Characterization of *numts*

**Fig. 1.**
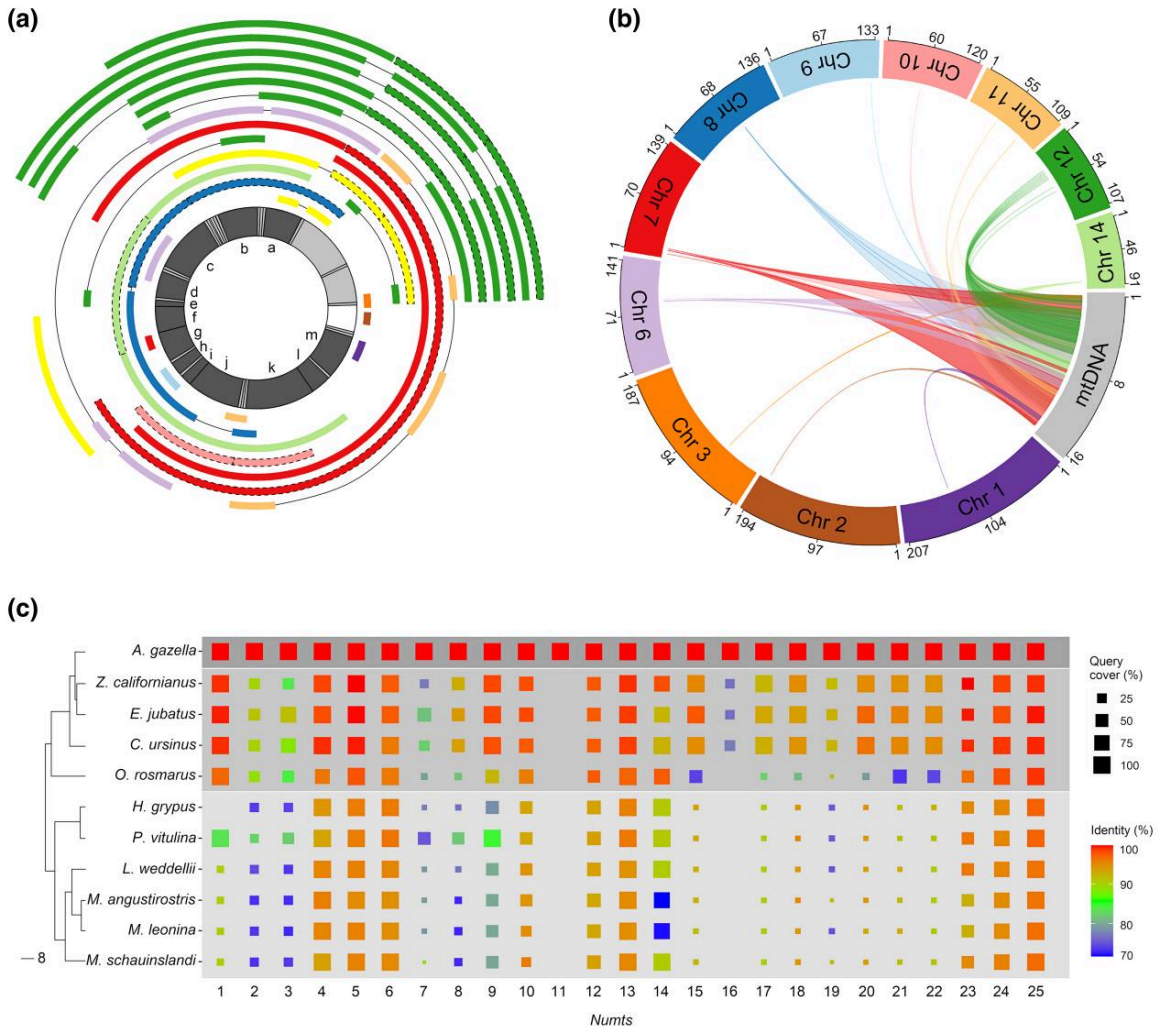
Summary of 25 *numts* characterized in *A. gazella* including their mitochondrial origins, locations in the nuclear genome, and patterns of phylogenetic conservation. (*a*) The mitochondrial origin of each *numt* (outer colored arcs). The innermost circle represents the *A. gazella* mitochondrial genome, where light grey sectors refer to tRNA and rRNA genes, dark grey sectors represent other mitochondrial genes (a: ND1, b: ND2, c: COX1, d: COX2, e: ATP8, f: ATP6, g: COX3, h: ND3, i: ND4L, j: ND4, k: ND5, l: ND6, m: CYTB) and the white sector represents the D-loop region. *Numts* are color coded according to the nuclear chromosome in which they are located as shown in (*b*). Yellow *numts*, absent in (*b*), were found in unplaced genomic scaffolds. Fragments belonging to the same *numt* are connected by solid black lines, whereas fragments belonging to the same *numt* and originating from overlapping mitochondrial regions are indicated with dashed lines. (*b*) The location of the *numts* in the *A. gazella* nuclear genome. Each colored arc connects the relevant region of the mitochondrial genome (grey) to the corresponding chromosome in the nuclear genome. Numbers indicate base-pair positions along the mitochondrial genome (kb) and along the nuclear chromosomes (Mb). (*c*) The results of blast searches against the nuclear genomes of *A. gazella* and other pinniped species (*Z. californianus*, *E. jubatus*, *C. ursinus*, *O. rosmarus*, *H. grypus*, *P. vitulina*, *L. weddellii*, *M. angustirostris*, *M. leonina*, and *M. schauinslandi*), whose phylogenetic relationships are indicated by the tree on the left (redrawn from Higdon et al., 2007). Each column represents a single *numt*. The colored squares indicate both the query cover (which is proportional to the size of square, see caption) and the percentage of sequence identity (indicated by the color gradient, see caption) when a given *numt* was blasted against the nuclear genome of another species. The circular plots were drawn using the R package circlize (Gu et al. 2014).

Sequence homology searches between the mitochondrial (Nagel et al. 2019) and nuclear genome (Peart et al. 2021) of the Antarctic fur seal reveal a total of 25 putative *numts* varying in length between 294 and 14,199 bp and consisting of between one and four separate fragments (see Materials and Methods, fig. 1 and table 1). These are located on 11 different chromosomes and three unplaced genomic scaffolds, each of which carries between one and eight *numts* (fig. 1*b* and table 1). The majority of these *numts* contain multiple full-length mitochondrial genes, and collectively they represent the entire mitochondrial genome (fig. 1*a*). The first 5,000 bp of the mitochondrial genome shows the highest average depth of coverage, reflecting the presence of multiple closely related *numts* on chromosome 12 (fig. 1*a*).

**Table 1:** Summary Information for All 25 *numts* Characterized in the Antarctic Fur Seal Nuclear Genome

Numt ID	Nuclear Scaffold	Start	End	Chromosome	Number of Fragments	Numt Length	mtDNA Coordinates	Expected PCR Product
1	ScWAj4l_1;HRSCAF = 3	127934607	127935176	9	1	569	9,566–10,139	Yes
2	ScWAj4l_2;HRSCAF = 13	74612525	74621691	8	4	9166	2,107–5,497; 7,736–10,840; 5,454–7,653; 11,628–12,127	Yes
3	ScWAj4l_18;HRSCAF = 239	84876333	84889827	14	2	13494	3,018–13,905; 6,301–8,931	No
4	ScWAj4l_37;HRSCAF = 538	184440549	184440843	2	1	294	15,767–16,061	Yes
5	ScWAj4l_43;HRSCAF = 587	54190362	54194849	10	2	4487	9,871–11,748; 11,733–13,078	Yes
6	ScWAj4l_44;HRSCAF = 590	2316056	2316387	7	1	331	8,723–9,062	No
7	ScWAj4l_44;HRSCAF = 590	7718809	7728008	7	2	9199	1–2,801; 10,088–16,156	No
8	ScWAj4l_44;HRSCAF = 590	7977588	7991731	7	4	14,143	9,436–16,156; 1–1,616; 2,738–6,806; 1,597–2,721	Yes
9	ScWAj4l_46;HRSCAF = 602	45289204	45289714	11	1	510	11,396–11,907	Yes
10	ScWAj4l_46;HRSCAF = 602	85808165	85817409	11	4	9,244	104–428; 1,757–2,257; 11,770–12,369; 14,387–15,304	Yes
11	ScWAj4l_49;HRSCAF = 609	50930992	50931325	3	1	333	1–326	Yes
12	ScWAj4l_82;HRSCAF = 715	87894123	87902946	6	4	8,823	3,942–5,516; 2,319–3,843; 10,185–10,984; 9,671–9,955	Yes
13	ScWAj4l_82;HRSCAF = 715	88838208	88839332	6	1	1,124	6,301–7,449	Yes
14	ScWAj4l_84;HRSCAF = 721	138510899	138511378	1	1	479	14,905–15,381	Yes
15	ScWAj4l_133;HRSCAF = 810	20791	26530	Unplaced scaffold	3	5,739	2,968–5,471; 1,597–2,721; 74–1,616	Yes
16	ScWAj4l_2350;HRSCAF = 3197	2	2125	Unplaced scaffold	2	2,123	2,177–2,819; 3,037–3,535	No
17	ScWAj4l_2441;HRSCAF = 3292	24002125	24007610	12	3	5,485	74–1,616; 2,968–4,007; 5,120–5,471	Yes
18	ScWAj4l_2441;HRSCAF = 3292	24086242	24091805	12	3	5,563	74–1,616; 2,738–5,471; 1,597–2,721	Yes
19	ScWAj4l_2441;HRSCAF = 3292	24285509	24299708	12	3	14,199	74–1,198; 2,738–5,471; 6,202–6,875	Yes
20	ScWAj4l_2441;HRSCAF = 3292	24425761	24432730	12	3	6,969	74–1,616; 2,968–6,806; 1,597–2,671	Yes
21	ScWAj4l_2441;HRSCAF = 3292	24605035	24611940	12	3	6,905	2,968–6,806; 74–1,198; 1,597–2,671	Yes
22	ScWAj4l_2441;HRSCAF = 3292	24665417	24670984	12	3	5,567	79–1,616; 2,740–5,471; 1,597–2,721	Yes
23	ScWAj4l_2441;HRSCAF = 3292	53287344	53289309	12	2	1,965	1,917–2,215; 78–432	No
24	ScWAj4l_2441;HRSCAF = 3292	60968082	60971080	12	2	2,998	3,905–4,582; 7,807–8,060	Yes
25	ScWAj4l_4416;HRSCAF = 5370	222	1965	Unplaced scaffold	1	1,743	8,175–9,955	Yes

Note.— Locations in the *A. gazella* nuclear genome are reported together with information on the number of *numt* fragments, total length, mitochondrial location of origin, and whether PCR amplification using nuclear genome-specific primers yielded a product of the expected size.

### Experimental Validation

PCR amplification using nuclear genome-specific primers yields PCR products of the approximate expected size in the majority of cases (*n* = 20, 80%, supplementary fig. S1, Supplementary Material online). Three of the PCR products are larger than expected, whereas a further two primer pairs yield unclear products with the suggestion of multiple bands (supplementary fig. S1, Supplementary Material online). The fact that most of the *numts* can be validated in vitro suggests that most if not all of them are genuine mitonuclear integrations rather than genome assembly artifacts.

### Phylogenetic Patterns

Blast searches of the 25 *numts* against available pinniped reference genomes produce highly variable profiles in terms of query cover and percentage of identity (fig. 1*c*). We interpret blast results characterized by high query cover as hits to homologous genomic regions and blast results showing low query cover as hits to nonhomologous regions. In fact, even in the case of highly diverged *numts* characterized by low percentage of identity, we would expect query cover to be relatively high also in the presence of gaps, as these can be identified during a blast search. The patterns summarized in figure 1*c* are then suggestive of an ongoing process of mitonuclear integration. Specifically, we find that some of the *numts* appear to have been integrated into the nuclear genome of the Antarctic fur seal rather recently, as these are unique to this species (e.g., *numts* 11 and 16, fig. 1*c*). These represent insertions that took place not earlier than the split between *Arctocephalus* species and *Zalophus californianus* and *Eumetopias jubatus* (fig. 1*c*), which occurred around 5.2 Ma. By contrast, other *numts*, which are shared among all of the pinniped species (e.g. *numts* 5 and 25, fig. 1*c*), are likely to represent ancient insertions that occurred before the split between the three pinniped families and are thus at least 25 Myr old (Deméré et al. 2003; Higdon et al. 2007). Finally, *numts* present in a subset of the pinniped species (e.g. *numts* 1 and 9, fig. 1*c*) were probably integrated at intermediate time points. Repeating this analysis separately for each *numt* fragment yields comparable results (supplementary fig. S2, Supplementary Material online). Moreover, this analysis also reveals a highly consistent pattern of query cover and percentage of identity among fragments clustered within the same *numt* (supplementary fig. S2, Supplementary Material online), suggesting that they can be considered part of the same mitonuclear insertion.

### Numt Coding Genes

To understand the evolutionary origins and fates of the identified mitonuclear insertions, we focus on *numt* fragments showing sequence homology to mitochondrial protein-coding DNA. Because we are specifically interested in genes showing intact mitochondrial reading frames, we focus on the separate *numt* fragments, as opposed to whole *numts*, in order to exclude the nuclear sequences located between *numt* fragments. We identify six *numt* fragments containing a total of 21 genes that contain intact mitochondrial reading frames (table 2), although all of them contain premature stop codons as defined by the nuclear genetic code. The majority of these genes are identical or nearly identical to their mitochondrial homologs and are therefore likely to represent recent integrations from the *A. gazella* mitochondrial genome. However, we also identify fragments in *numts* 1, 2, and 3 that contain genes differing by more than five nucleotide substitutions from their mitochondrial homologs. Pairwise dN/dS analysis of genes located in these *numt* fragments reveal strong signatures of purifying selection, indicated by dN/dS < 1 (table 2). Synonymous substitution rates (dS), a proxy for neutral divergence, for the fragments in *numts* 2 and 3 are modest, indicating that the substitutions observed at these genes are likely to have resulted from relatively recent insertions of highly divergent mtDNA sequences, as compared with the Antarctic fur seal reference mitochondrial genome. By contrast, dS for the gene located in *numt* 1, ND3, exceeds one (table 2), indicating that this *numt* shows substantial neutral divergence at synonymous sites relative to its mitochondrial homolog.

**Table 2:** The Number of Synonymous and Nonsynonymous Substitutions, Together with Relative dN and dS Measures, Between Protein-Coding Genes Residing in the *numt* Fragments and Their mtDNA Homologs

Numt	Fragment	Gene	Accession Number	Synonymous Substitutions	Nonsynonymous Substitutions	dN	dS	dN/dS	*P*-value
1	1	ND3	DAC80253.1	48	14	0.0564	1.1062	0.051	<0.01
2	1	ATP8	DAC80250.1	0	0	—	—	—	—
2	1	ATP6	DAC80251.1	0	0	—	—	—	—
2	1	COX3	DAC80252.1	0	0	—	—	—	—
2	1	ND3	DAC80253.1	0	0	—	—	—	—
2	1	ND4L	DAC80254.1	1	0	—	0.0148	0	—
2	3	ND2	DAC80247.1	46	16	0.0218	0.1881	0.1160	<0.01
3	1	COX1	DAC80248.1	0	1	0.0009	—	—	—
3	1	ATP8	DAC80250.1	0	0	—	—	—	—
3	1	COX3	DAC80252.1	13	4	0.0066	0.0835	0.0794	<0.01
3	1	ND3	DAC80253.1	5	2	0.0075	0.0719	0.1042	<0.01
3	1	ND4L	DAC80254.1	4	0	—	0.0683	0	—
3	1	ND4	DAC80255.1	24	5	0.0052	0.0615	0.085	<0.01
3	1	ND5	DAC80256.1	30	5	0.0038	0.0667	0.0562	<0.01
3	2	COX2	DAC80249.1	7	1	0.0019	0.0472	0.0404	<0.01
3	2	ATP8	DAC80250.1	0	1	0.0068	—	—	—
3	2	ATP6	DAC80251.1	4	5	0.0103	0.022	0.4668	0.2145
8	3	ND4L	DAC80254.1	4	0	—	0.0683	0	—
8	3	ND4	DAC80255.1	0	0	—	—	—	—
8	3	ND5	DAC80256.1	0	0	—	—	—	—
8	3	CYTB	DAC80258.1	0	0	—	—	—	—

Note.—Identities and NCBI accession numbers are provided for each gene.

### Phylogenetic Analysis of Fragments in *numt* 1, 2, and 3

We implement phylogenetic analysis of the fragments in *numts* 1, 2, and 3 that contain genes with intact reading frames in order to better understand when these insertions might have colonized the Antarctic fur seal nuclear genome. Our phylogenetic reconstruction is based on the Antarctic fur seal *numt* fragments and on homologous sequences from the mtDNA of *A. gazella*, 26 other pinniped species (see Materials and Methods) and the dog (*Canis lupus*). We additionally include sequences of homologous *numts* from other pinnipeds when present. Consistent with our previous results, *numt* 1 appears to be a relatively old insertion (supplementary fig. S3*a*, Supplementary Material online) that predates the split between the walrus and the otariids, which occurred around 18 Ma (Higdon et al. 2007), whereas *numts* 2 and 3 appear to be more recent (supplementary fig. S3*b* and *c*, Supplementary material online). Notably, the *numt* 2 sequences from *E. jubatus*, *Z. californicus*, *Callorhinus ursinus*, and *Odobenus rosmarus* cluster separately and more distantly from the *numt* 2 sequence of *A. gazella*, suggesting that these sequences may not be homologous (supplementary fig. S3*b*, Supplementary material online). For *numt* 3, we only analyzed the nuclear sequence of *A. gazella* because we could not identify homologous sequences in other pinnipeds (blast hits were obtained to multiple locations with low query cover and percentage of identity).

### Selection Analysis of *numt* 1

**Fig. 2.**
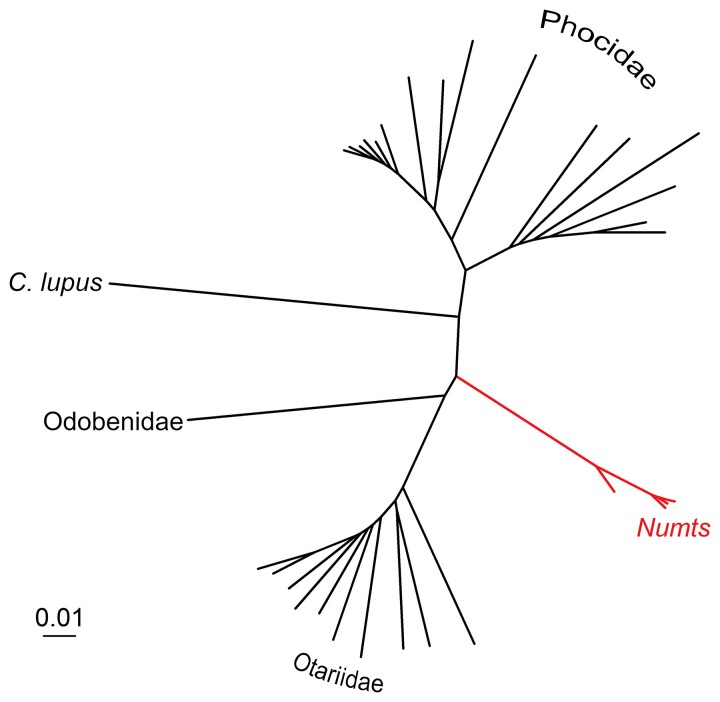
Phylogenetic reconstruction of *numt* 1. The phylogenetic tree was built using the nuclear sequences of *numt 1* found in *A. gazella*, *Z. californianus*, *E. jubatus*, *C. ursinus*, and *O. rosmarus* together with homologous mtDNA sequences from all pinnipeds with an available mitochondrial reference genome (see Materials and Methods). The branch containing the nuclear sequences is indicated in red.

Phylogenetic analysis of *numt* 1 suggests that this integration likely occurred around 18 Ma (fig. 2). We therefore wanted to know if the mitochondrial reading frames of the gene in *numt* 1 are also conserved in other pinnipeds, which is indeed the case. We investigated the possibility of a potential functional conservation of *numt* 1 on the protein-coding level since it was integrated into the nuclear genome by conducting substitution rate analyses to identify the evolutionary forces that have shaped the sequence composition of the gene ND3 located in *numt* 1 (indicated by the red branch in fig. 2). By estimating dN/dS ratios for the predefined branches (red vs. black, fig. 2), we find that dN/dS ratios are significantly different between the *numt* 1 gene sequences and their mitochondrial counterparts (*P* < 0.001, likelihood ratio test [LRT], one-ratio vs. two-ratio model, table 3). This pattern cannot be solely attributed to differences in the intensity of purifying selection (*P* = 0.99, LRT, two-ratio vs. two-ratio fixed model, table 3). Instead, neutral evolution and/or positive selection may have played a pervasive role in the evolution of *numt* 1.

**Table 3:** Summary Statistics of the Substitution Rate Ratio Models Implemented to Test for Functional Conservation of the ND3 Gene Located in *numt* 1

Model	Site Class	Proportion	dN/dS (mitochondria)	dN/dS (NUMTs)	LogL
One-ratio	/	/	0.01613		−1828.804375
Two-ratio	/	/	0.01105	0.99424	−1811.746294
Two-ratio (fixed)	/	/	0.01105	1	−1811.746326
Clade C	1	0.57666	0	0	−1802.187057
	2	0	1	1	
	3	0.42334	0.02947	2.7993	
Clade C (fixed)	1	0.55786	0	0	−1803.236605
	2	0	1	1	
	3	0.44214	0.02856	1	
M1A	1	0.97937	0.02063		−1827.229958
	2	0.02063	1		
M22	1	0.90499	0.00806		−1815.988374
	2	0	1		
	3	0.09501	0.13246		

Note.—For each model, we report dN/dS ratios for mitochondrial and nuclear sequences separately, as well as the associated log likelihood value. Models “Clade C,” “Clade C (fixed),” “M1A,” and “M22” take into account heterogeneity of the dN/dS ratio across the protein sequence and the sites are therefore partitioned into three classes. Site class 1 corresponds to sites under purifying selection (dN/dS < 1), site class 2 corresponds to neutral sites (dN/dS = 1), and site class 3 corresponds to unrestricted sites.

To test this, we refined our model by accounting for heterogeneity in the dN/dS ratio across the protein sequence, which is a realistic assumption for mammalian proteins (Afanasyeva et al. 2018). Specifically, we assume that differences in the dN/dS ratio between the *numt* 1 genes and their mitochondrial counterparts can be attributed to differences in selection pressures on particular codons. A model that incorporates such differences (model Clade C) fits the data significantly better than branch models (*P* < 0.001, LRT, Clade C vs. two-ratio, table 3) as well as site models (*P* < 0.001, LRT, Clade C vs. M1a and LRT, Clade C vs. M22, *P* < 0.001, table 3), illustrating that part of the *numt* 1 coding sequences are evolving under strong purifying selection (site class 1, dN/dS = 0). Interestingly, the Clade C model estimates that positive selection also plays a role (dN/dS = 2.8 for class 2, table 3), although this is marginally nonsignificant when compared with a Clade C model that assumes site class 2 to be evolving under drift (*P* = 0.15, LRT, Clade C vs. Clade with fixed site class 2: dN/dS = 1, table 3).

### Sequence Context of *numt* 1 in other Pinnipeds

To better understand the functional context of the genomic regions surrounding *numt* 1, we extract 150 kb of flanking sequences from each side of this *numt* from all five Otaroidea species where the *numt* is present (with the exception of the walrus, where only ∼110 kb of sequence is available on the 5′ side of the *numt*). Blastx searches against the available mammalian genome sequences in the NCBI nonredundant database reveal that in all five species, the genomic regions flanking *numt* 1 are enriched with retrotransposable elements (supplementary fig. S4, Supplementary Material online). Specifically, we retrieve several partial hits to the ORF2p gene of *C. lupus* (accession number: QOV08757.1) in four species. Top hits in the walrus are to the LORF gene of *Crocuta crocuta* (accession number: KAF0872869.1) and to the endonuclease/reverse transcriptase of *Sus scrofa* (accession number: ABR01162.1). Furthermore, the genomic regions surrounding *numt* 1 appear to have a very similar organization in at least four of the pinnipeds (supplementary fig. S4, Supplementary Material online), suggesting that these regions are homologous. This is harder to evaluate for the walrus due to the smaller number of retrieved annotations and the fact that these produce hits to different species.

## Discussion

To shed light on the origin and evolution of nuclear copies of mitochondrial genes, we searched for the presence of mitochondrial sequences in the nuclear genome of the Antarctic fur seal, identifying a total of 25 *numts*. By focusing on mitonuclear insertions containing genes whose mitochondrial reading frames are intact, we identified one potentially functional *numt* and two mitonuclear fragments that represent good candidates for noncanonical sources of mitonuclear integrations. The first clearly represents an ancient insertion that we can characterize in homologous genomic regions across five divergent pinniped species and that appears to have been subject to strong purifying selection since the time of insertion. By contrast, the other two mitonuclear insertions represent recent integrations that likely originate from the integration of heteroplasmic mtDNA or as a result of hybridization.

Because their integration into eukaryotic cells, mitochondrial genomes have substantially reduced in size, as their functional genes were transferred into the nuclear genome (Bock and Timmis 2008). Our results are consistent with the notion that this is a continuous and still ongoing process (Ricchetti et al. 2004), given that we found evidence for *numts* having originated at different time points in the evolutionary history of pinnipeds. On the one hand, certain *numts* were present in all of the investigated species and may therefore be more than 25 Myr old, which is when pinnipeds diverged from the other arctoid carnivores (Deméré et al. 2003; Higdon et al. 2007). On the other hand, some of the *numts* were only found in the Antarctic fur seal, meaning that they were probably integrated into this species’ nuclear genome sometime during the past 5.2 Myr.

Nuclear copies of mitochondrial genes have been characterized in a variety of mammals (Ning et al. 2017; Schiavo et al. 2017). With its 25 *numts* comprising a total of 57 separate fragments, the Antarctic fur seal is a species characterized by a relatively low *numt* content, as the number of identified *numts* exceeds 100 in most of the species examined so far (Calabrese et al. 2017). Nevertheless, the *numts* that we identify collectively represent the entire *A. gazella* mitochondrial genome. In line with previous studies (Tsuji et al. 2012), we also found that different mtDNA regions are integrated to varying extents into the nuclear genome, with the D-loop region being the least represented.

We identified multiple *numts* spanning mitochondrial regions containing genes. However, based on the mitochondrial genetic code, most of them harbor premature stop codons and out of frame mutations, suggesting that they are ancient insertions. By contrast, fragments in *numts* 1, 2, 3, and 8 contain genes with intact mitochondrial reading frames. In *numt* 2 (only fragment 1) and 8, these are identical or nearly identical to their mitochondrial homologs and may represent very recent insertions that have not yet accumulated mutations. Given that the PCR amplification of these *numts* yielded a product of the expected size, we believe they are genuine recent insertions and not genome assembly artifacts. By contrast, the remaining genes in *numt* 1, 2 (only fragment 3), and 3 harbor substantially more substitutions and we therefore investigated them in greater detail.

*Numt* 1 is characterized by a surprisingly high synonymous substitution rate. Our phylogenetic analysis shows that this *numt* is an ancient insertion, which explains the high level of observed sequence divergence. Specifically, this *numt* appears to be present in homologous genomic regions across five pinnipeds belonging to the family Otaroidea. The ND3 gene in *numt* 1 contains an intact mitochondrial reading frame in all of these species and our substitution rate analyses reveals that it has undergone strong purifying selection since the time of insertion. Previous studies have shown that *numts* are unlikely to be transcribed (Bensasson et al. 2001). In line with this, we found that the ND3 gene in *numt* 1 contains premature stop codons as defined by the nuclear genetic code. It also appears doubtful that *numts* play a role in the generation of small mitochondrial RNAs (Pozzi and Dowling 2019), small noncoding RNAs of mitochondrial origin that have been suggested to exert a functional role, possibly via RNA interference (Cloonan 2015). Nonetheless, the fact that the ND3 gene in *numt* 1 deviates from expectations under selective neutrality suggests that it is likely to be somehow functional. Noutsos et al. (2007) showed that nuclear insertions of organellar DNA can create novel exons, but this was observed uniquely in plants. Moreover, such novel exons appeared to derive from markedly reshaped protein domains, which is not the case for *numt* 1. Thus, the most likely explanation for our findings is that *numt* 1 might have a function as regulatory element. An alternative but arguably less likely possibility could be that *numt* 1 inserted into a region of an ancestral Otaroidea genome characterized by a very low mutation rate, which would have prevented disruption of the mitochondrial reading frame. In this scenario, the large number of observed substitutions would have been caused by mutations in the mtDNA rather than in the *numt* itself. Such a *numt* would then represent a “genomic fossil,” which provides a picture of the sequence of the Otaroidea ND3 mitochondrial gene prior to the radiation of the Odobenidae and Otariidae. Regardless of the exact explanation for our findings, our study paves the way for future research aimed at understanding the functional role of *numts*.

Functional annotation of the flanking sequences of *numt* 1 across five Otaroid species shows that this *numt* is located in a genomic region rich in retrotransposable elements. Specifically, most of the hits we retrieved were to the ORF2p gene, an endonuclease of the LINE-1 retrotrasposon (Kines et al. 2016). This is not surprising as retrotransposable elements are frequently found in the flanking sequences of *numts* (Tsuzuki et al. 1983; Zullo et al. 1991; Tsuji et al. 2012). LINE-1 retrotransposons in particular are often overrepresented (Zullo et al. 1991; Tsuji et al. 2012) to the extent that Tsuji et al. (2012) speculated that the LINE-1 endonuclease might play a role in both retrotransposon and *numt* expansion.

*Numts* 2 and 3 appear to have resulted from more recent insertion events, as their sequences cluster closely to the *A. gazella* mitochondrial homologs in our phylogenetic reconstructions. Nevertheless, the genes in these *numts* show high dissimilarity to the homologous *A. gazella* mitochondrial sequence, which poses the question of which mtDNA lineage(s) they originated from. One possibility is that they could have originated from a mitochondrial sequence belonging to a closely related pinniped species that introgressed into *A. gazella* as a result of hybridization. In support of this hypothesis, it is known that Antarctic fur seals hybridize with *A. tropicalis* and, to a lesser extent, with males of *A. forsteri* (Lancaster et al. 2006). Under this scenario, it is possible that a recent mitonuclear insertion that was already present in *A. tropicalis* or *A. forsteri* introgressed into the *A. gazella* nuclear genome as a consequence of hybridization. Alternatively, introgressed mtDNA haplotypes might have been inserted into the *A. gazella* nuclear genome after hybridization. However, both hypotheses cannot yet be formally tested as mitochondrial reference genomes are currently lacking for *A. tropicalis* and *A. forsteri*. Alternatively, these *numts* may represent mitonuclear insertions of highly divergent *A. gazella* heteroplasmic mtDNA variants (Formenti et al. 2021; Parakatselaki and Ladoukakis 2021). These could potentially derive from the inheritance of paternal mtDNA that, despite occurring rarely, has been documented in humans (Wei et al. 2020) and other vertebrates (Kvist et al. 2003; Zhao et al. 2004). In support of both of these hypotheses, the genes in *numts* 2 and 3 appear to be under purifying selection, which probably reflects the fact that until recently they were expressed as part of the mitochondrial genome. Finally, we cannot exclude the possibility that *numt* 3 (but not *numt* 2) could be a genome assembly artifact given that the PCR product for this *numt* was not of the expected size.

In conclusion, by focusing on functionally intact genes in *numts* and by characterizing their synonymous and nonsynonymous substitution rates, we uncovered the presence of a potentially functional *numt* in the Antarctic fur seal genome. Future research is needed to ground truth the functionality of the nuclear version of the ND3 gene and to better understand how this functionality may be exerted. We also identified two mitonuclear insertions that do not appear to originate from the mitochondrial genome DNA of the Antarctic fur seal and which probably represent insertions from either heteroplasmic mtDNA variants or mtDNA introgressed via hybridization. Taken together, our findings reveal not only the promise of our approach for better understanding the origin and evolution of *numts*, but also the potential of mitonuclear integrations as genomic fossils to pinpoint historic mitochondrial variation.

## Materials and Methods

### Numt Discovery

We searched for *numts* using the methodology of Lammers et al. (2017). Briefly, the complete mitochondrial genome of the Antarctic fur seal (Nagel et al. 2019) (NCBI accession number: BK010918) was searched against the nuclear reference genome of the same species (Peart et al. 2021) (NCBI accession number: GCA_900642305.1) using BLAST (Camacho et al. 2009) with a word_size of 20 bp. Matching sequences were then filtered for length and only those longer than 200 bp were classified as putative *numts* and retained for further analysis. Multiple mitochondrial sequence fragments that mapped to within 10 kb of one another in the nuclear genome are clustered into a single *numt*. This was done to avoid artificially inflating the number of detected *numts*, as many of these fragments are likely to be part of the same mitonuclear insertions.

### Experimental Validation

We attempted to experimentally validate the presence of all 25 *numts* in the Antarctic fur seal nuclear genome by designing PCR primers specific to the nuclear genome. Because many of these *numts* are very large, we did not attempt to PCR amplify full-length sequences. Instead, we designed one primer inside of the *numt* and the other primer either outside of the *numt* or overlapping the boundary between the *numt* and the adjacent nuclear genome. We used in silico PCR to check that none of the primer pairs would amplify a region of the mitochondrial genome. PCR amplification was consequently expected to yield a product only if both of the primers annealed to the nuclear genome. Primer design was implemented using default parameters in PRIMER3 (Untergasser et al. 2007).

Genomic DNA was extracted from two Antarctic fur seal individuals using a standard chloroform/isoamylalcohol extraction protocol (Sambrookand et al. 1989). PCR amplification with each primer pair was undertaken using a Type-it microsatellite PCR kit (QIAGEN) with a temperature profile of initial melting for 5 min at 95 °C, followed by 40 amplification cycles of 30 s at 95 °C, 30 s at 56 °C, and 50 s at 72 °C, and concluding with a elongation step of 30 min at 60 °C. An established primer pair targeting the MC1R gene was included as positive control (Peters et al. 2016). PCR products were visualized on a 2% agarose gel.

### Phylogenetic Characterization of *numts*

In order to shed light on the timings of the identified mitonuclear integrations, we carried out blast searches of the *numt* sequences characterized in the Antarctic fur seal against the nuclear genomes of other pinniped species. Specifically, we focused on all ten species for which a nuclear reference genome is currently available (*Z. californianus*, GCF_009762345.2; *E. jubatus*, GCA_004028035.1; *C. ursinus*, GCA_003265705.1; *O. rosmarus*, GCA_000321225.1; *Halichoerus grypus*, GCA_012393455.1; *Phoca vitulina*, GCA_004348235.1; *Leptonychotes weddellii*, GCA_000349705.1; *Mirounga angustirostris*, https://www.dnazoo.org/assemblies/Mirounga_angustirostris; *M. leonina*, GCA_011800145.1; and *Monachus schauinslandi*, GCA_002201575.1). Blast searches were carried out separately for each species using blastn default parameters and an *E*-value cutoff of 0.01. For each *numt*, we recorded the percentage of query cover and the percentage of identity of the top hit to characterize its similarity to the corresponding *A. gazella numt*. This analysis was additionally repeated considering all *numt* fragments separately. This was done with the aim of verifying whether different mitonuclear fragments clustered within the same *numt* can be considered part of the same mitonuclear insertion.

### Identification of Potential Protein-Coding Numts

Pairwise alignments between the sequences of 13 genes present in the *A. gazella* mitochondrial genome (Nagel et al. 2019) and each separate *numt* fragment were performed to identify *numt* fragments containing genes with intact mitochondrial reading frames. Gap-free alignments where the respective *numt* fragment sequence did not contain a premature stop codon were extracted. Pairwise nonsynonymous (dN) and synonymous (dS) substitution rates were then calculated using KaKs Calculator v2.0 (Wang et al. 2010) to assess the magnitude of divergence between the nuclear and mitochondrial homologs.

### Comparative Substitution Rate Analysis

To better understand the evolutionary forces that shaped the observed sequence divergence, we carried out pairwise dN/dS analyses (Kryazhimskiy and Plotkin 2008) between the mitochondrial genes and their respective homologous in the relevant *numt* fragments. Specifically, dN/dS ratios close to one indicate the random fixation of mutations. By contrast, ratios below one are indicative of purifying selection, whereas ratios above one are suggestive of the presence of beneficial mutations.

### Phylogenetic Analysis of *numts* Containing Potential Coding Genes

High dS values (>1) along with low dN values (<<1) could result from historic *numt* integrations. We therefore implemented phylogenetic analyses of *numts* showing this signature to better understand their possible origin(s). To do so, we first retrieved homologous *numt* sequences from other pinniped nuclear genomes using BLAST. Second, we identified homologous mitochondrial sequences from other pinniped species by BLASTing the *A. gazella numt* sequences against the complete mitochondrial genomes of 26 pinniped species (*A. australis*, MG023139.1; *A. forsteri*, NC_004023.1; *A. townsendi*, NC_008420.1; *A. pusillus*, NC_008417.1; *C. ursinus*, NC_008415.3; *Cystophora cristata*, NC_008427.1; *Erignathus barbatus*, NC_008426.1; *E. jubatus*, NC_004030.2; *H. grypus*, NC_001602.1; *P. fasciata*, NC_008428.1; *P. groenlandica*, KP942529.1; *P. larga*, NC_008430.1; *P. vitulina*, NC_001325.1; *Hydrurga leptonyx*, NC_008425.1; *L. weddellii*, NC_008424.1; *Lobodon carcinophaga*, NC_008423.1; *M. leonina*, NC_008422.1; *M. monachus*, NC_044972.1; *M. schauinslandi*, NC_008421.1; *Neophoca cinerea*, NC_008419.1; *Phocarctos hookeri*, NC_008418.1; *Pusa caspica*, NC_008431.1; *P. hispida*, NC_008433.1; *P. sibirica*, NC_008432.1; *O. rosmarus*, NC_004029.2; *Z. californianus*, NC_008416.1) as well as the dog (*C. lupus familiaris*, NC_002008.4) using a word_size of 20 and an *E*-value cutoff of 0.01. Next, we aligned the pinniped *numts* and the corresponding mitochondrial sequences of the Antarctic fur seal, the other 26 pinnipeds species and the dog. This was implemented using the MUSCLE algorithm in MEGA (Kumar et al. 2018). Finally, phylogenetic trees were constructed within MEGA as neighbour-joining trees using the Tajima-Nei model method, which allows for the mutation rate to be heterogeneous among different lineages (Nei and Kumar 2000).

### Analysis of Functional Conservation of the ND3 Gene Located on *numt* 1

Genes located in an ancient mitonuclear insertion showing signatures of purifying selection might reveal evidence of functional conservation at the protein-coding level since their integration into the nuclear genome. We therefore investigated whether the gene located on *numt* 1, ND3, shows signals of such functional conservation across the Otaroidea. To do so, we divided the branches of the phylogeny constructed as described above into one set of branches forming the clade consisting of all *numt* sequences since the integration into the nuclear genome, and one set consisting of the rest of the tree (fig. 2, red and black branches, respectively). We then performed a series of substitution rate ratio model comparisons using codeml (Yang 2007), where different substitution rates on coding DNA and estimates of dS were used for nuclear and mitochondrial branches to account for the difference in mutation rates between the mitochondrial and the nuclear genome. We implemented a series of branch models (Yang 1998; Yang and Nielsen 1998). First, we assumed a model with a single dN/dS (“one-ratio model”) over the entire phylogeny. Second, we assumed free dN/dS for the two branch classes (“two-ratio model”) and third, we implemented a model where we fixed the dN/dS for the *numt* clade to 1, but kept the other class free (e.g. neutral evolution in the *numt* clade, “two-ratio model fixed”). Finally, we conducted likelihood ratio tests to check whether the models with varying dN/dS between the two branch classes (e.g. two-ratio models) provide a better fit to the data than the model with a single dN/dS or a model of neutral evolution. Next, to account for heterogeneity of selective pressures within the protein sequence, we implemented branch-site models, using the same branch classification as above (*numt* branches vs. the rest of the tree), but allowing three site classes (class 1: dN/dS < 1, class 2: dN/dS = 1, and site class 3: unrestricted). We implemented the Clade model C (Bielawski and Yang 2004), where only site class 2 can be different between the branch classes. The Clade C model was compared with site models M1A and M22 using LRTs, as suggested by the PAML user manual.

### Annotation of numt 1 Flanking Sequences

The flanking sequences of *numt* 1, which is believed to represent an ancient insertion that underwent strong purifying selection, were extracted from all of the Otaroidea reference genomes and annotated. This had the double aim of checking whether this *numt* was present in homologous genomic regions and of functionally characterizing the DNA sequences surrounding it. Specifically, we extracted 150 kb of flanking sequences from each side of the *numt* and blasted these against the nonredundant NCBI database, whereas limiting our searches to mammals. The top hit of each search was then used for annotation.

## Supplementary Material

Supplementary materials are available at *Genome Biology and Evolution* online (http://www.gbe.oxfordjournals.org/).

## Supplementary Material

evac104_Supplementary_Data

## Data Availability

All of the data analyzed in this study are already publicly available. The characterized *numt* sequences have been deposited in figshare (https://figshare.com/articles/dataset/Antarctic_fur_seal_NUMTs_sequences_SPP_1158_/16399938, keyword: SPP 1158). The code relevant for this study is available at: https://github.com/DavidVendrami/Antarctic_fur_seal_numts.
