# Supplementary material for: Signatures of Selection on Mitonuclear Integrated Genes Uncover Hidden Mitogenomic Variation in Fur Seals
Source: Genome Biol Evol. 2022 Jul 9;14(7):evac104. doi: 10.1093/gbe/evac104 (PMC9338431; doi:10.1093/gbe/evac104)
Supplement: evac104_Supplementary_Data [file evac104_supplementary_data.pdf]

Supplementary Information for

Signatures of selection on mitonuclear integrated genes uncover hidden mitogenomic variation in fur seals

David L. J. Vendrami[1], Toni I. Gossmann[1 †], Nayden Chakarov[1†], Anneke J. Paijmans[1], Adam Eyre-Walker[2], Jaume Forcada[3], & Joseph I. Hoffman[1,3,*]

1. Department of Animal Behaviour, Bielefeld University, 33501 Bielefeld, Germany
2. School of Life Science, University of Sussex, Brighton, BN1 9QG
3. British Antarctic Survey, High Cross, Madingley Road, Cambridge CB3 OET, UK

† These authors contributed equally

*** Corresponding author**:
Joseph. I. Hoffman, Department of Animal Behaviour, Bielefeld University, 33501 Bielefeld, Germany, +49 521 106 2711, joseph.hoffman@uni-bielefeld.de

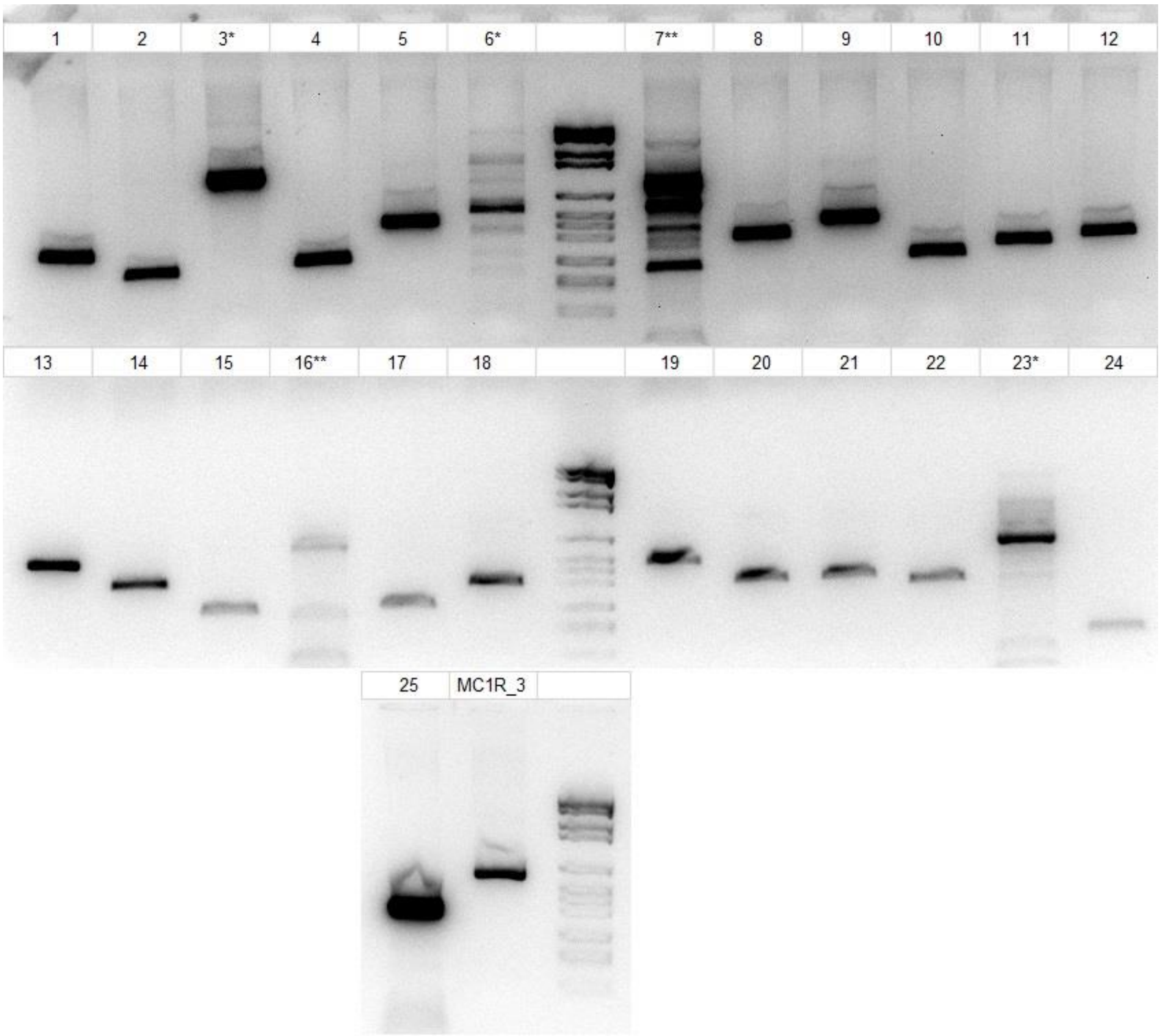

Supplementary Figure 1. Gel electrophoresis of the PCR amplification products obtained for 25 *numts*. The central lane contains the pBR328 marker (Carl Roth). A fragment of the melanocortin-1 receptor gene (denoted MC1R_3) was PCR amplified as positive control. *Numts* that yielded longer than expected products are indicated with a star (*), while *numts* yielding multiple electrophoresis bands are indicated with two stars (**).

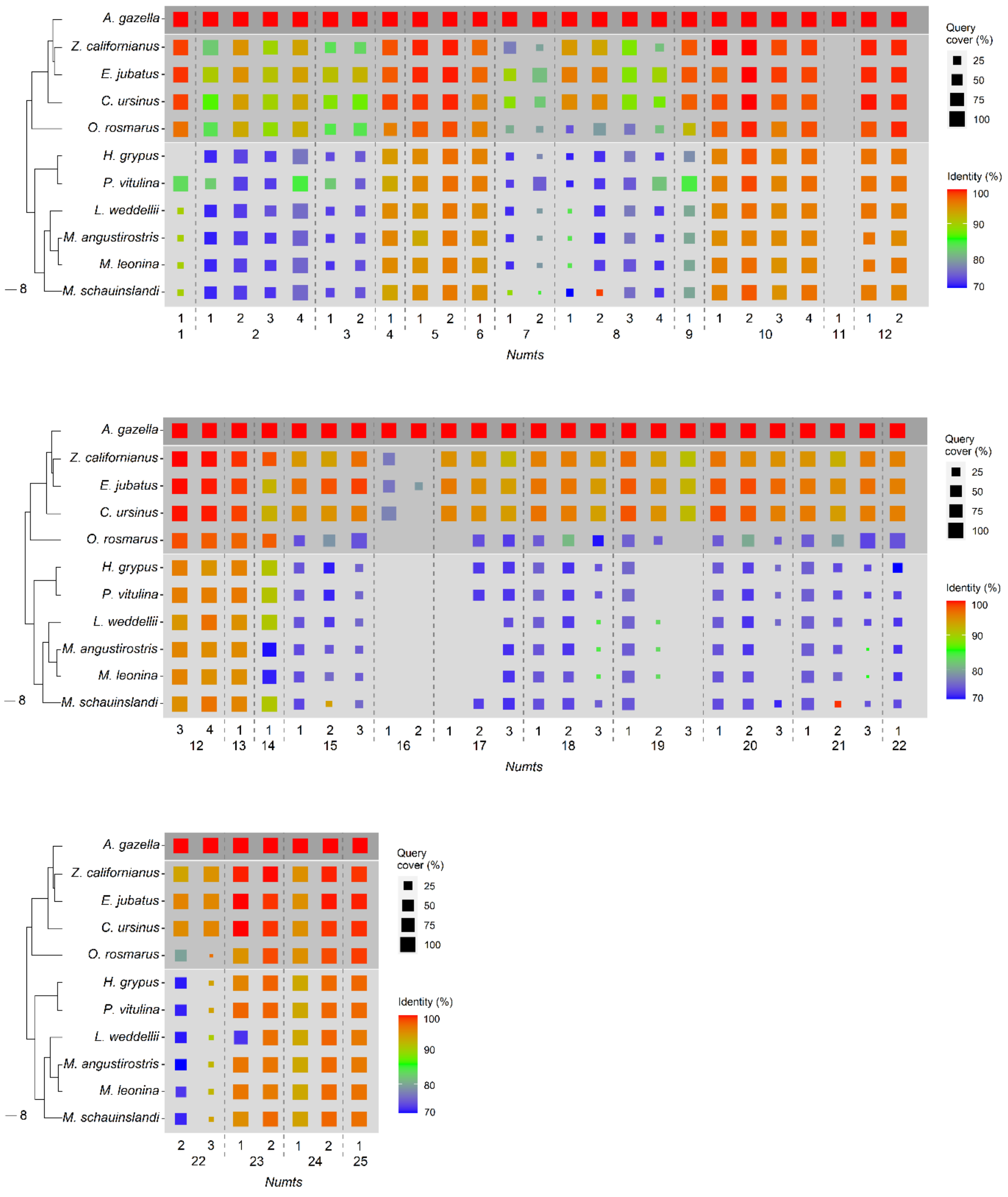

Supplementary figure 2. Patterns of phylogenetic conservation across the 57 identified *numt* fragments. Shown are the results of blast searches against the nuclear genomes of *A. gazella* and other pinniped species (*Z. californianus*, *E. jubatus*, *C. ursinus*, *O. rosmarus*, *H. grypus*, *P. vitulina*, *L. weddellii*, *M. angustirostris*, *M. leonina* and *M. schauinslandi*), whose phylogenetic relationships are indicated by the tree on the left (redrawn from[37]). Each column represents a single *numt* fragment. Vertical dashed lines separate *numt* fragments belonging to the same *numt*. Each coloured square indicates both the query cover (which is proportional to the size of square, see caption) and the percentage of sequence identity (indicated by the colour gradient, see caption) when a given *numt* fragment was blasted against the nuclear genome of another species.

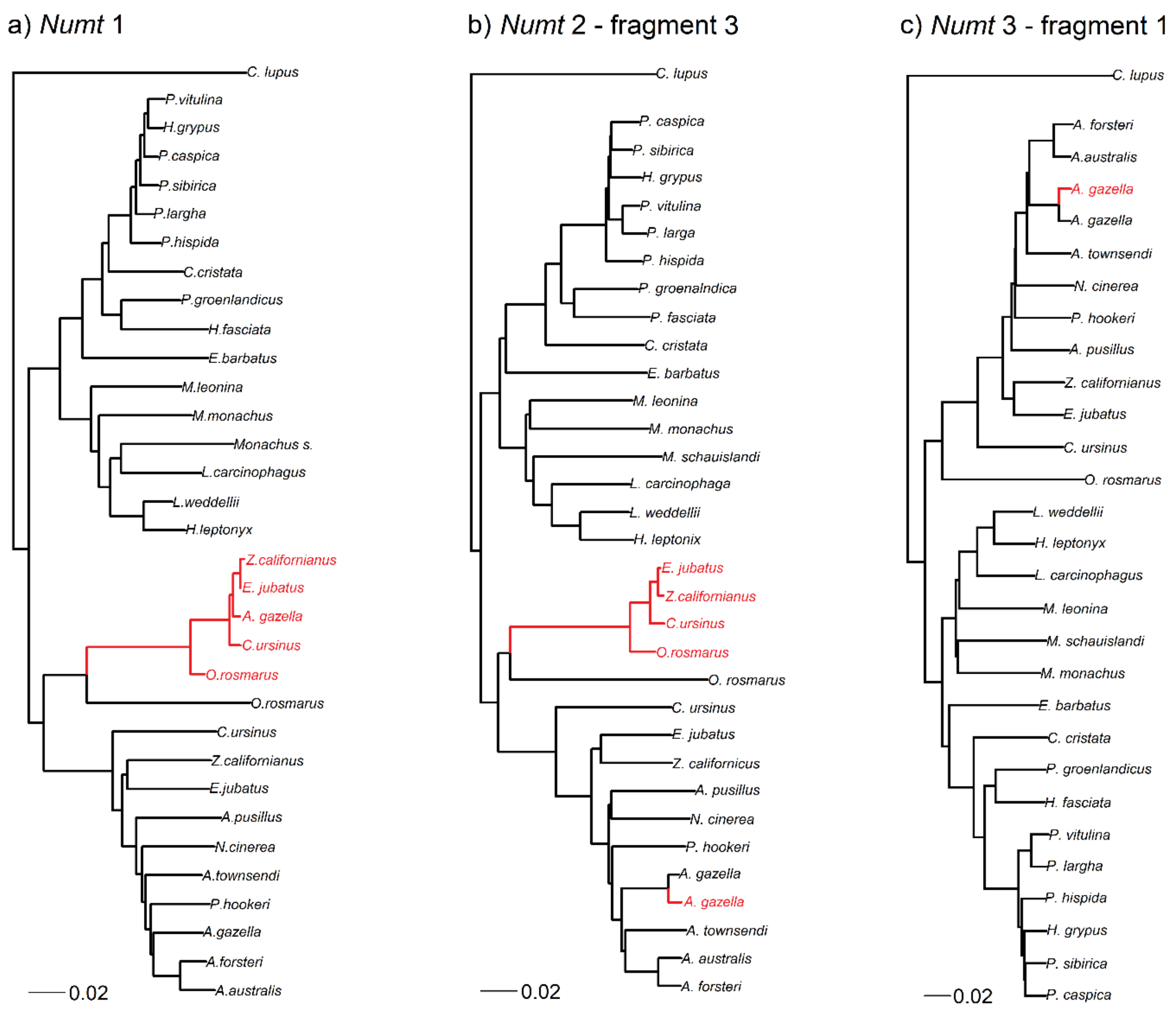

Supplementary Figure 3. Phylogenetic trees constructed using the *numt* fragments sequences (red branches) and the mitochondrial DNA homologs from *A. gazella* and other pinniped species (black branches). The phylogenetic trees integrating *numt* 1, 2 (fragment 3) and 3 (fragment 1) are shown in panels a), b) and c) respectively.

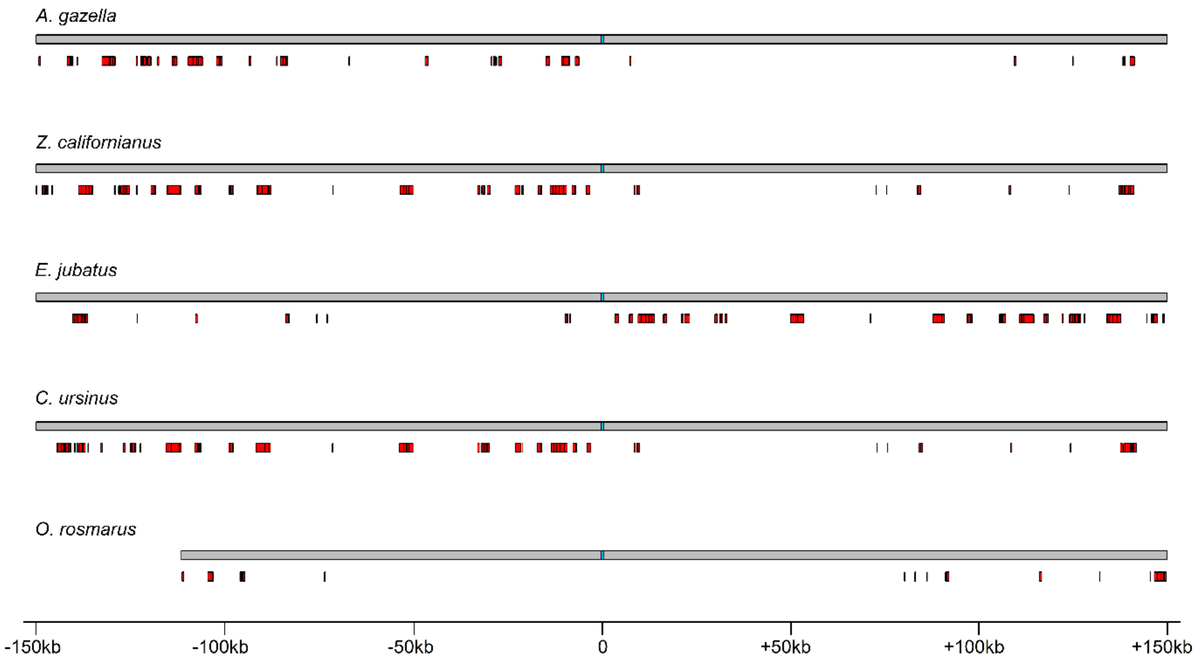

Supplementary Figure 4. Genomic location of *numt* 1 in five Otaroidea species, together with blastx hits for its flanking sequences. Shown are the genomic regions spanning 150kb on each side of *numt* 1 (light blue rectangle at position zero) in the genomes (grey) of the Antarctic fur seal (*A. gazella*), the California sea lion (*Z. californianus*), the Northern sea lion (*E. jubatus*), the Northern fur seal (*C. ursinus*) and the Walrus (*O. rosmarus*). Red rectangles represent blastx top hits. These are to the ORF2p [*C. lupus*] gene in *A. gazella*, *Z. californianus*, *E. jubatus* and *C. ursinus* and to the LORF [*C. crocuta*] gene and endonuclease/reverse transcriptase [*S. scrofa*] gene in *O. rosmarus*. This region appears to be inverted in *E. jubatus*.
